# Child With Human Herpesvirus 6 and Bell’s Palsy: Case Report and Overview of the Literature

**DOI:** 10.7759/cureus.84563

**Published:** 2025-05-21

**Authors:** Loren Adler, Avery Schroeder, Paul Carney

**Affiliations:** 1 Medical School, University of Missouri School of Medicine, Columbia, USA; 2 Pediatrics and Neurology, University of Missouri School of Medicine, Columbia, USA

**Keywords:** bell's palsy, hhv-6, peripheral facial nerve palsy, roseola, viral reactivation

## Abstract

Peripheral facial nerve palsy (pFP), or Bell’s palsy, does occur in the pediatric population, although it is rare. Numerous infectious etiologies of pFP are known, but up to 75% of pediatric pFP cases are idiopathic. Empiric treatment typically involves steroids and antivirals, and etiologic testing is not frequently conducted. Human herpesvirus 6 (HHV-6) has been reported as a possible cause of pFP in both adults and children. We report a case of a 17-month-old female who presented with pFP and had a history of HHV-6 infection five months prior to presentation. This case may represent another instance of HHV-6 as a potential causative agent of pFP. While it cannot be stated with certainty, this finding lends itself to the consideration of HHV-6 as a cause of pFP, both in the setting of acute infection and viral reactivation.

## Introduction

Peripheral facial nerve palsy (pFP), or Bell’s palsy, is a rare occurrence, especially in the pediatric population, in which the reported incidence is 6.1 per 100,000 per year in patients aged one to 15 years. Bell's palsy presents as sudden, unilateral facial weakness, presenting as ptosis and inability to smile or express on that side of the face. It is caused by a peripheral insult to the seventh cranial nerve, the facial nerve, which leads to upper and lower unilateral motor and sensory deficits on the face. It is self-limited, but typically steroids and/or antivirals are given to support recovery. The majority of patients with pFP regain their function fully within nine months. Up to 70% of adult pFP cases and 75% of pediatric cases are idiopathic [1,2], but commonly identified infectious etiologies generally involve the herpes virus family [3-7]. Human herpes virus 6 (HHV-6) is an umbrella term encompassing HHV-6A and HHV-6B, both of which are members of the Roseolovirus genus of the Betaherpesviridae subfamily [8]. HHV-6B is the cause of roseola infantum or exanthema subitum and is much more prevalent, while HHV-6A is less well-understood and more frequently affects immunocompromised patients [8]. Over 95% of humans over the age of two years are seropositive with either or both variants [8]. HHV-6 can be asymptomatic but is usually associated with undifferentiated fever. The clinical manifestations are termed roseola infantum or exanthema subitum when there is high fever up to 40°C that lasts for three to five days, followed by a blanching maculopapular rash that spreads centrifugally. The diagnosis is clinical [9]. HHV-6 has been shown to establish life-long latent infections, and reactivation in immunocompromised hosts has been linked to encephalitis, multiple sclerosis, and other neurological diseases [9]. 

HHV-6 specifically has been hypothesized as a potential etiology for pFP, both in children and adults, as HHV-6 has been found in CSF, serum, saliva, and tear samples of patients with pFP [3,7,10]. HHV-6 has also been found in facial nerve ganglia, CSF, serum, saliva, and tear samples of patients without pFP [11-13], and some studies have noted that the prevalence and viral load of HHV-6 in these groups is higher than in control groups [3,4,7,10]. It has been shown that herpes simplex virus 1 (HSV-1) is capable of remaining latent and later reactivating in facial nerve ganglia, leading to the suggestion that HHV-6 and other herpesviruses may be capable of the same phenomenon as it relates to pFP [5]. There is currently no consensus in the literature regarding whether HHV-6 is a definitive causative agent of pFP, but there have been multiple cases reported of concurrent HHV-6 infection and pFP [14,15] as well as cases of HHV-6 infection associated with other cranial nerve palsies [16,17]. Several studies have suggested a role for HHV-6 in pFP, but the presence of a causative relationship is not universally agreed upon [3,4,11,17]. Other articles have concluded that there is no evidence for HHV-6 as a causative agent, nor evidence to support the hypothesis of viral reactivation in general as an etiology of pFP [13,18]. The case presented here offers an additional report of a possible relationship between HHV-6 and pFP. 

## Case presentation

We report the case of a 17-month-old female who presented to pediatric neurology clinic for follow-up of pFP diagnosed 16 days prior in the emergency department (ED). The patient's right-sided facial drooping was first noted by her mother on the evening prior to ED presentation. At this time, the facial droop was so mild that the patient's mother was unsure whether the child was intentionally distorting her face or exhibiting genuine abnormality. The following morning, the facial droop was severe, prompting the patient to be brought to the ED.

**Figure 1:**
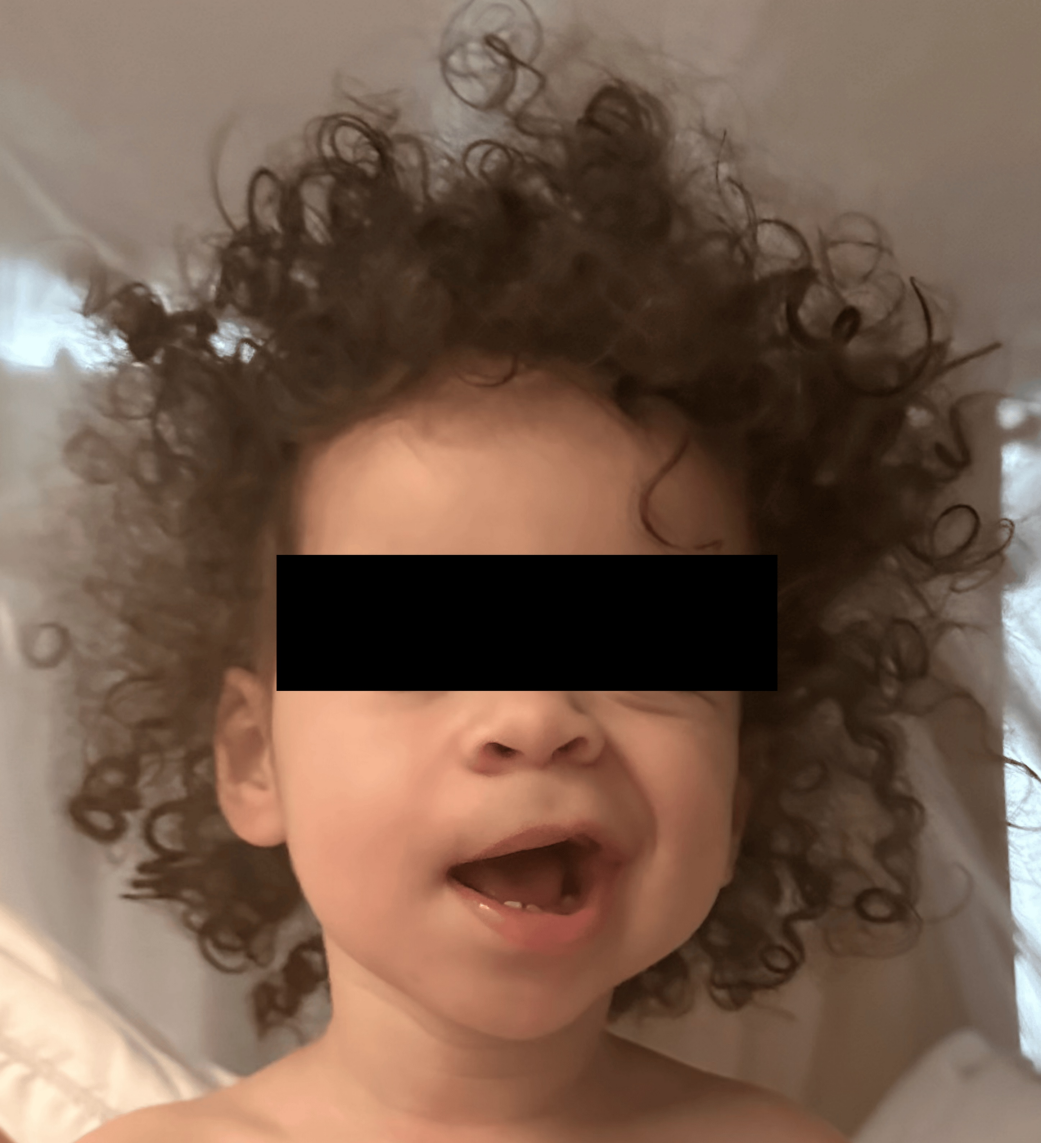
Patient photo, demonstrating paralysis of right upper and lower face (photo taken with cellphone front camera, shortly prior to ED presentation). Photo utilized and published with consent from the parent.

On evaluation at the ED, the patient was noted to have right-sided facial paralysis (Figure 1) and a bulging, non-erythematous tympanic membrane on the right side, ruling down severe otitis media as an etiology of the symptoms. No other abnormalities were noted on physical exam, and vital signs were within normal limits. No herpetic vesicles or other rash were noted anywhere on the patient’s body. No labs or imaging were performed during ED evaluation. The patient was diagnosed with idiopathic peripheral facial nerve palsy and discharged from the ED after receiving a 5 mg of oral dexamethasone. She was sent home and completed a seven-day course of 200 mg acyclovir three times daily (for coverage of possible HSV-1 infection) and a second dose of 6 mg of dexamethasone four days after the first. Interestingly, while no labs were ordered during her ED evaluation, the patient had a standing order for CBC (placed previously by her primary care physician) that was drawn two days after ED presentation. This was remarkable for a mild leukocytosis to 11.76x109 and thrombocytosis to 521x109 (Table 1). 

**Table 1:** Results of laboratory studies performed two days after patient presentation to the emergency department. WBC = white blood cell count; RBC = red blood cell count; HGB = hemoglobin; PLT = platelet count

	TEST	RESULT	REFERENCE
HEMATOLOGY	WBC (x10^9^/L)	11.76	6-11
	RBC (x10^12^/L)	4.44	3.7-6
	HGB (g/dL)	11.5	10.5-13.5
	PLT (x10^9^/L)	521	150-450

On presentation to clinic 17 days after symptom onset, the patient’s facial weakness was almost completely resolved. The patient had some lingering weakness around the right side of the mouth, with her smile remaining slightly asymmetric, but strength around the eyelid and eyebrow was markedly improved. It was further questioning at this clinic visit which yielded the fact that the patient was clinically diagnosed with HHV-6 infection five months prior to pFP onset, after being seen in the clinic for fever with a maximum temperature of 102.6°F (39.2°C) and developing a characteristic HHV-6 rash days later. Additionally, she had experienced three days of upper respiratory tract infection symptoms which resolved spontaneously 14 days prior to her pFP onset, but no pathogen was identified as no care was sought. 

## Discussion

While the current literature offers no consensus regarding the existence of a role for HHV-6 in the pathogenesis of peripheral facial nerve palsy, the case presented here may lend some additional support to this hypothesis [13,18]. This child presented with a unilateral pFP in a non-Lyme-endemic region with a history of HHV-6 infection five months prior to symptom onset and upper respiratory tract infection two weeks prior to symptom onset. The patient was vaccinated against varicella-zoster virus (VZV) and had no rash or skin lesions at the time of presentation, ruling down the likelihood of HSV and VZV as etiologic agents. However, absence of lesions cannot rule out HSV-1 and the empiric treatment of pFP with acyclovir is often performed without confirmation of HSV-1 infection [1]. Differentiation of etiologies without definitive evidence or characteristic signs can be quite difficult. This patient’s presentation overall does not favor any specific etiology of pFP, however the relatively recent history of HHV-6 and the suggested pathogenesis of reactivation does offer a possible link between the infection and the pFP presentation.

The association between HHV-6 and pFP has a reasonable degree of scientific basis, given the virus’s familial relationship with herpes simplex virus, which has been shown to cause pFP in response to both novel infection and viral reactivation [3,5,6]. This, in combination with the evidence that HHV-6 can remain latent in the salivary gland, in the facial nerve ganglion, and in neurons and glia, lends itself to the consideration that HHV-6 may offer some potential for causation in pFP pathogenesis [3,12,19]. Performing HHV-6 polymerase chain reaction (PCR) testing in patients with peripheral facial nerve palsy may shed additional light on this potential relationship [14,20]. Doing so may eventually allow diagnosticians of pFP to limit prescribing medications like acyclovir, which is commonly given empirically to cover presumed HSV involvement, when it might not be indicated due to a specific identified etiology [1].

## Conclusions

While it is impossible to be certain of whether HHV-6 played a role in this patient’s pFP presentation, it serves as an additional case to reinforce the possibility of a role for HHV-6 in pFP pathogenesis. If nothing else, this warrants future consideration of HHV-6 as a possible etiology for pFP in children.
